# Implementation of the 2017 American College of Cardiology/American Heart Association Guidelines on Hypertension in Clinical Practice

**DOI:** 10.31486/toj.20.0105

**Published:** 2021

**Authors:** Poonam Mahato, Rajan Ganesh, Balaram Krishna Hanumanthu, Yesha Patel Rana, Jin Ei Chan, Tahmina Alam, Deepika Misra

**Affiliations:** ^1^ Department of Internal Medicine, Division of Cardiology, Mount Sinai Beth Israel/Icahn School of Medicine at Mount Sinai, New York, NY; ^2^ Department of Internal Medicine, Mount Sinai Beth Israel/Icahn School of Medicine at Mount Sinai, New York, NY

**Keywords:** *Compliance*, *guideline adherence*, *health plan implementation*, *hypertension*

## Abstract

**Background:** The 2017 American College of Cardiology (ACC)/American Heart Association (AHA) guidelines on hypertension recommend a threshold blood pressure (BP) of ≥130/80 mmHg for diagnosis of hypertension and treating hypertension to a goal BP of <130/80 mmHg. For this study, we assessed the rate of compliance to the 2017 ACC/AHA hypertension guidelines by internal medicine residents and cardiology fellows in clinics affiliated with a teaching hospital in New York, New York.

**Methods:** We conducted a retrospective medical records review for patients who had a clinical encounter at the internal medicine resident and cardiology fellow clinics from January to February 2019. To distinguish from adherence with prior guidelines, patients with BP of 130-139/80-89 mmHg (unless age ≥60 years and systolic blood pressure [SBP] 140-149 mmHg without chronic kidney disease or diabetes) were included. The primary outcome was accurate assessment of uncontrolled BP in accordance with the 2017 ACC/AHA guidelines.

**Results:** Included in the analysis were 435 patients from the internal medicine resident clinic and 127 patients from the cardiology fellow clinic. Accurate assessment of uncontrolled BP was higher in the cardiology fellow clinic compared to the internal medicine resident clinic (29.1% vs 10.3%, *P*<0.001), even after adjusting for baseline characteristics differences between the 2 clinics. Multivariate regression analysis revealed that the type of clinic (internal medicine, odds ratio [OR] 0.27, 95% CI 0.16-0.47; *P*<0.001), established diagnosis of hypertension (OR 2.06, 95% CI 1.06-3.99; *P*<0.001), and SBP (OR 1.16 per mmHg, 95% CI 1.11-1.22; *P*=0.031) were independently associated with the primary outcome.

**Conclusion:** Cardiology fellows were better at identifying hypertension diagnosis thresholds and BP treatment goals in accordance with 2017 ACC/AHA guidelines compared to internal medicine residents.

## INTRODUCTION

The Seventh Joint National Commission Report (JNC 7) published in 2003 defined hypertension as systolic blood pressure (SBP) ≥140 mmHg or diastolic blood pressure (DBP) ≥90 mmHg and recommended treating blood pressure (BP) to a goal BP of <140/90 mmHg in the general population and <130/80 mmHg in patients with diabetes or chronic kidney disease (CKD).^1^ The Eighth Joint National Commission Report (JNC 8) published in 2014 revised the BP treatment goals to <140/90 mmHg for adults <60 years of age, <150/90 mmHg for adults ≥60 years of age, and <140/90 mmHg for adults with diabetes or CKD, regardless of age.^2^ The 2017 American College of Cardiology/American Heart Association (ACC/AHA) hypertension guidelines^3^ incorporated information from more recent studies, especially the SPRINT (Systolic Blood Pressure Intervention Trial) trial,^4^ to establish stricter cutoffs for the diagnosis of hypertension and the treatment goals compared to the JNC guidelines. In summary, the ACC/AHA defined hypertension as SBP ≥130 mmHg or DBP ≥80 mmHg based on ≥2 readings obtained on ≥2 occasions. BP goal was <130/80 mmHg for all adults. Pharmacologic therapy was recommended for anyone with BP ≥140/90 mmHg and for patients with BP 130-139/80-89 mmHg and established atherosclerotic cardiovascular disease (ASCVD) or an ASCVD 10-year risk of ≥10%.

A review of the literature revealed a few small studies^5^ that have reported variable rates of physician adherence to the JNC 7 and JNC 8 guidelines on hypertension.^1,2^ Between May 2012 and April 2013, a cross-sectional descriptive survey investigated 59 primary care physicians working in 33 primary health centers in the Aljouf region of the Kingdom of Saudi Arabia and found that 80% adhered to the JNC 7 guidelines on hypertension.^6^ From February 2004 to October 2004, a retrospective medical records review of 345 patients evaluated physician adherence to the JNC 7 guidelines on hypertension using 22 criteria in 6 community-based clinics in Iowa and concluded that general adherence was 53.5%.^7^ A 2007 retrospective review of 251 medical records at West Virginia University, Charleston, found an overall BP goal achievement of 45.6% by internal medicine residents.^8^

Studies focusing on the 2017 ACC/AHA hypertension guidelines have primarily evaluated the change in incidence and prevalence rates of newly diagnosed hypertension.^9^ Our review of the literature showed a lack of studies assessing the implementation of the new guidelines in clinical practice. Specifically, to the authors’ knowledge, the implementation of the 2017 ACC/AHA hypertension guidelines based on the physicians’ levels of training has not been explored.

For this study, we assessed the rate of implementation of the hypertension diagnosis thresholds and treatment goals defined by 2017 ACC/AHA hypertension guidelines at the internal medicine resident and cardiology fellow clinics affiliated with a teaching hospital in a major city. We also explored the hypertension management practices by internal medicine residents and cardiology fellows.

## METHODS

We reviewed the medical records of patients who had a clinical encounter at the internal medicine resident clinic and cardiology fellow clinic associated with Mount Sinai Beth Israel, a teaching hospital in New York, New York. The study periods for the internal medicine resident and cardiology fellow clinics were January 1 to January 31, 2019, and January 1 to February 28, 2019, respectively. The additional month for the cardiology fellow clinic study period was included to help offset the greater volume of patients seen in the internal medicine resident clinic.

Patients with recorded SBP ≥130 mmHg or DBP ≥80 mmHg during the clinical encounter were included in the study regardless of established diagnosis of hypertension. To avoid conflict with adherence to prior guidelines, patients whose BP would be considered appropriate by JNC 8^2^ guidelines were excluded from the study. Hence, the study included (1) all patients with DBP 80-89 mmHg, (2) all patients with SBP 130-139 mmHg, and (3) patients with SBP 140-149 mmHg if they were ≥60 years of age without a history of CKD or diabetes. To reduce observer bias, the patients who were seen in the clinic by the investigators of this study were also excluded.

The clinical progress notes from the encounters were reviewed, and the patients’ demographic information (age, sex, body mass index, tobacco abuse), BP, relevant medical history, medication list, and the parameters to calculate ASCVD 10-year risk^10^ were noted. SBP and DBP were assessed as normal, elevated in isolation, or elevated in combination. Definitions of isolated SBP elevation (SBP ≥130 mmHg and DBP <80 mmHg) and isolated DBP elevation (SBP <130 mmHg and DBP ≥80 mmHg) were used to identify and differentiate these phenotypes. Serum cholesterol levels were noted from laboratory results obtained within 6 months prior to the clinic encounter. ASCVD 10-year risk percentage was estimated for patients aged 40 to 79 years with adequate data in accordance with the calculator from the ACC website.^11^ Patients with established ASCVD—defined as coronary artery disease, prior myocardial infarction, peripheral artery disease, or cerebrovascular disease—were excluded from ASCVD 10-year risk calculation in accordance with recommendations.

For the purposes of this study, patients with uncontrolled BP included patients with known hypertension and BP not at goal (ie, SBP ≥130 mmHg or DBP ≥80 mmHg) and patients who presented for their initial encounter with BP that would be considered in the hypertensive range (ie, SBP ≥130 mmHg or DBP ≥80 mmHg) and who needed reassessment at future visits to confirm hypertension.

The primary outcome of the study was defined as the correct assessment of uncontrolled BP by physicians in accordance with the 2017 ACC/AHA guidelines. Physicians were noted to have correctly assessed uncontrolled BP if they fulfilled the following criteria: (1) For patients with an established diagnosis of hypertension, they mentioned in their clinic note that SBP ≥130 mmHg or DBP ≥80 mmHg was above goal, regardless of the intervention done; and (2) for patients without an established diagnosis of hypertension, they mentioned in their clinic note that SBP ≥130 mmHg or DBP ≥80 mmHg was elevated and would need to be reassessed.

The secondary outcome was defined as the type of intervention done once uncontrolled BP was correctly assessed. Interventions included prescribing a home BP monitor; providing advice on lifestyle changes, including the Dietary Approaches to Stop Hypertension (DASH) diet^12^; prescribing new antihypertensives; increasing the dose of existing medication; reviewing and reconciling medications; placing a consult to a specialist/hypertension nurse; and reassessing BP during the next visit.

Baseline characteristics were analyzed and summarized using descriptive statistics: mean ± SD for continuous parametric variables, median and interquartile range for nonparametric data, and frequency (percentage) for categorical or nominal variables. Baseline continuous variables were compared between the 2 groups using *t* test or nonparametric equivalent, and chi-square test (Fisher exact test in the case of sparse data) was used to compare categorical and nominal variables. Outcomes were assessed with logistic regression adjusting for risk factors described later in the text. Statistical significance was defined as a *P* value of ≤0.05. Data were analyzed using Stata, release 13 (StataCorp).

Approval for the study was granted by the institutional review board of the Mount Sinai School of Medicine. The authorization for use and disclosure of personal health information was waived because of minimal harm.

## RESULTS

We reviewed 1,175 and 405 charts from the internal medicine resident and cardiology fellow clinics, respectively. After application of inclusion and exclusion criteria, 435 (37.0%) patients from the internal medicine resident clinic and 127 (31.4%) patients from the cardiology fellow clinic were included for analysis (n=562).

The baseline characteristics of the patients seen in the 2 clinics are displayed in Table 1. The mean ages of the patients seen at the cardiology fellow clinic and the internal medicine resident clinic were similar (57 years vs 59 years, *P*=0.19). The proportion of female patients was higher in the internal medicine resident clinic compared to the cardiology fellow clinic (69.4% vs 48.0%, *P*<0.001). History of hypertension (73.2% vs 58.9%, *P*=0.003) and established ASCVD (29.1% vs 14.7%, *P*<0.001) were higher among patients seen in the cardiology fellow clinic vs the internal medicine resident clinic. Congestive heart failure (15.7% vs 3.7%, *P*<0.001) and atrial fibrillation (10.2% vs 3.7%, *P*=0.003) were also more prevalent among patients seen in the cardiology fellow clinic vs the internal medicine resident clinic. Average SBP was higher among patients from the cardiology fellow clinic vs the internal medicine resident clinic (132.5 ± 6.7 mmHg vs 131.0 ± 7.3 mmHg, *P*=0.042), whereas average DBP was lower for patients from the cardiology fellow clinic vs the internal medicine resident clinic (78.9 ± 7.7 mmHg vs 80.8 ± 5.9 mmHg, *P*=0.004). The cardiology fellow clinic had a higher percentage of patients with isolated SBP elevation than the internal medicine resident clinic (37.0% vs 24.8%, *P*=0.007) and a lower percentage of patients with isolated DBP elevation than the internal medicine resident clinic (25.2% vs 36.1%, *P*=0.022). Use of any antihypertensives (68.5% vs 56.8 %, *P*=0.014), beta blockers (44.9% vs 15.6%, *P*<0.001), angiotensin converting enzyme inhibitors (ACEi)/angiotensin receptor blockers (ARB)/angiotensin receptor neprilysin inhibitors (ARNi) (54.3% vs 40.0%, *P*=0.004), aspirin (42.5% vs 32.0%, *P*=0.027), and statins (63.0% vs 49.7%, *P*=0.008) was also higher among patients seen in the cardiology fellow clinic compared to patients seen in the internal medicine resident clinic. Notably, body mass index, active tobacco abuse, history of diabetes, CKD stage ≥3, cholesterol levels, and 10-year ASCVD risk for patients without established ASCVD were not statistically different between the 2 patient groups.

**Table 1.** Baseline Characteristics of Patients by Clinic

Variable	Cardiology Fellow Clinic, n=127	Internal Medicine Resident Clinic, n=435	*P* Value
Age, years, mean ± SD	57 ± 14	59 ± 15	0.19
Female	61 (48.0)	302 (69.4)	<0.001
Black/African American^a^	30/123 (24.4)	71/430 (16.5)	0.045
Body mass index, kg/m^2^, mean ± SD	30.6 ± 7.1	29.8 ± 6.7	0.25
Tobacco use			0.34
Never smoker	72 (56.7)	252 (57.9)	
Former smoker	45 (35.4)	132 (30.3)	
Current smoker	10 (7.9)	51 (11.7)	
Comorbidities			
Diagnosed hypertension	93 (73.2)	256 (58.9)	0.003
Coronary artery disease	33 (26.0)	46 (10.6)	<0.001
Peripheral artery disease	1 (0.8)	6 (1.4)	0.6
Cerebrovascular accident	7 (5.5)	23 (5.3)	0.92
Diabetes mellitus	36 (28.3)	131 (30.1)	0.7
Chronic kidney disease	6 (4.7)	40 (9.2)	0.11
Congestive heart failure	20 (15.7)	16 (3.7)	<0.001
Atrial fibrillation	13 (10.2)	16 (3.7)	0.003
Obstructive sleep apnea	7 (5.5)	15 (3.4)	0.29
Blood pressure			
SBP, mmHg, mean ± SD	132.5 ± 6.7	131.0 ± 7.3	0.042
DBP, mmHg, mean ± SD	78.9 ± 7.7	80.8 ± 5.9	0.004
Isolated SBP elevation^b^	47 (37.0)	108 (24.8)	0.007
Isolated DBP elevation^c^	32 (25.2)	157 (36.1)	0.022
Lipid panel			
LDL, mg/dL, mean ± SD	101.0 ± 43.3	108.9 ± 42.0	0.078
HDL, mg/dL, mean ± SD	50.2 ± 16.2	51.5 ± 14.8	0.42
Total cholesterol, mg/dL, mean ± SD	175.9 ± 50.4	184.1 ± 48.1	0.11
Antihypertensive use	87 (68.5)	247 (56.8)	0.014
Thiazide	16 (12.6)	70 (16.1)	0.34
Beta-blocker	57 (44.9)	68 (15.6)	<0.001
Calcium channel blocker	32 (25.2)	91 (20.9)	0.31
ACEi/ARB/ARNi	69 (54.3)	174 (40.0)	0.004
Aspirin	54 (42.5)	139 (32.0)	0.027
Statin	80 (63.0)	216 (49.7)	0.008
Atherosclerotic cardiovascular disease (ASCVD)			
Established ASCVD	37 (29.1)	64 (14.7)	<0.001
ASCVD 10-year risk, %^d^			0.26
<10	30 (23.6)	156 (35.9)	
≥10	29 (22.8)	109 (25.1)	
Unable to calculate^e^	31 (24.4)	106 (24.4)	0.144
ASCVD 10-year risk, %, median [IQR]	9.8 (4.7, 16.1)	8.6 (4.1, 16.4)	0.57

^a^Race data could not be confirmed for 4 patients in the cardiology fellow clinic and 5 patients in the internal medicine resident clinic.

^b^Isolated SBP elevation, patients with SBP ≥130 mmHg and DBP <80 mmHg.

^c^Isolated DBP elevation, patients with SBP <130 mmHg and DBP ≥80 mmHg.

^d^ASCVD 10-year risk was only calculated for people without established ASCVD.

^e^Unable to calculate ASCVD because of age <40 years, age >79 years, inadequate data including lipid panel and race information, and total cholesterol <130 mg/dL or >320 mg/dL.

Note: Data are presented as n (%) unless otherwise noted.

ACEi, angiotensin-converting enzyme inhibitor; ARB, angiotensin receptor blocker; ARNi, angiotensin receptor neprilysin inhibitor; ASCVD, atherosclerotic cardiovascular disease; DBP, diastolic blood pressure; HDL, high-density lipoprotein; IQR, interquartile range; LDL, low-density lipoprotein; SBP, systolic blood pressure.

Accurate assessment of uncontrolled BP in accordance with ACC/AHA 2017 guidelines—the primary outcome of the study—occurred more frequently in the cardiology fellow clinic than the internal medicine resident clinic (29.1% vs 10.3%, *P*<0.001) (Table 2). The occurrence rates for the assignment of individual interventions are also shown in Table 2. Counseling regarding lifestyle changes was less frequently performed in the cardiology fellow clinic than in the internal medicine resident clinic (0% vs 3.4%, *P*=0.034). Compared to patients from the internal medicine resident clinic, patients from the cardiology fellow clinic had higher rates of prescriptions for new antihypertensives (3.9% vs 1.1%, *P*=0.037), uptitration of the dose of antihypertensives (7.9% vs 0.7%, *P*<0.001), reconciliation of antihypertensives (3.9% vs 0.5%, *P*=0.002), and planned reassessment at the next visit (10.2% vs 3.0%, *P*<0.001). Differences in rates of home BP monitor prescriptions (3.9% vs 2.3%, *P*=0.31) and referral to a specialist (0% vs 0.7%, *P*=0.35) between cardiology fellow clinic patients and internal medicine resident clinic patients did not reach statistical significance.

**Table 2.** Study Outcomes by Clinic

Outcome	Cardiology Fellow Clinic, n=127	Internal Medicine Resident Clinic, n=435	*P* Value
Primary outcome			
Accurate assessment of uncontrolled blood pressure in accordance with 2017 ACC/AHA guidelines	37 (29.1)	45 (10.3)	<0.001
Secondary outcomes			
Home blood pressure monitor prescription	5 (3.9)	10 (2.3)	0.31
Lifestyle changes	0 (0.0)	15 (3.4)	0.034
New antihypertensive medication prescription	5 (3.9)	5 (1.1)	0.037
Uptitration of antihypertensives	10 (7.9)	3 (0.7)	<0.001
Antihypertensive medication reconciliation	5 (3.9)	2 (0.5)	0.002
Consult to cardiology/hypertension nurse	0 (0.0)	3 (0.7)	0.35
Reassessment at next visit	13 (10.2)	13 (3.0)	<0.001

Note: Data are presented as n (%).

ACC, American College of Cardiology; AHA, American Heart Association.

Logistic regression was performed to identify factors associated with the primary outcome (Table 3). Secondary outcomes were not adjusted for, given the low event rate. In univariate analysis, significant predictors associated with accurate assessment of uncontrolled BP included clinic type, age, established diagnosis of hypertension, SBP, isolated DBP elevation, any antihypertensive use, beta blocker use, calcium channel blocker use, and ACEi/ARB/ARNi use.

**Table 3.** Primary Outcome Logistic Regression Analyses

Variable	Univariate Analysis, Odds Ratio (95% CI)	*P V*alue	Multivariate Analysis, Odds Ratio (95% CI)	*P* Value
Internal medicine resident clinic	0.28 (0.17-0.45)	<0.001	0.27 (0.16-0.47)	<0.001
Age	1.02 (1.0-1.04)	0.004		
Female	0.73 (0.45-1.19)	0.216		
Black/African American	1 (0.54 - 1.84)	0.988		
Body mass index	0.99 (0.95-1.02)	0.683		
Active tobacco use	0.86 (0.6-1.23)	0.419		
Diagnosed hypertension	3.43 (1.88-6.28)	<0.001	2.06 (1.06-3.99)	<0.001
Coronary artery disease	1.6 (0.87-2.94)	0.127		
Peripheral artery disease	0.97 (0.11-8.2)	0.982		
Cerebrovascular accident	1.5 (0.59-3.79)	0.391		
Diabetes mellitus	0.73 (0.42-1.25)	0.255		
Chronic kidney disease	1.25 (0.56-2.8)	0.575		
Congestive heart failure	1.74 (0.76-3.97)	0.185		
Atrial fibrillation	0.93 (0.31-2.75)	0.901		
Obstructive sleep apnea	1.76 (0.63-4.93)	0.276		
SBP	1.17 (1.12-1.22)	<0.001	1.16 (1.11-1.22)	0.031
DBP	0.98 (0.95-1.02)	0.573		
Isolated SBP elevation	1.35 (0.81-2.23)	0.242		
Isolated DBP elevation	0.15 (0.06-0.33)	<0.001		
LDL	0.99 (0.99-1.00)	0.351		
HDL	1.00 (0.99-1.01)	0.589		
Antihypertensive use	2.54 (1.47-4.38)	0.001		
Thiazide	0.83 (0.42-1.65)	0.608		
Beta-blocker	2.33 (1.41-3.86)	0.001		
Calcium channel blocker	1.83 (1.09-3.07)	0.021		
ACEi/ARB/ARNi	2.32 (1.43-3.75)	0.001		
Aspirin	1.43 (0.88-2.3)	0.143		
Statin	1.24 (0.77-1.99)	0.362		
Established ASCVD	1.46 (0.83-2.57)	0.187		
ASCVD 10-year risk	1.02 (0.99-1.04)	0.069		
ASCVD ≥10%	1.82 (0.97-3.4)	0.059		

ACEi, angiotensin-converting enzyme inhibitor; ARB, angiotensin receptor blocker; ARNi, angiotensin receptor neprilysin inhibitor; ASCVD, atherosclerotic cardiovascular disease; DBP, diastolic blood pressure; HDL, high density lipoprotein; LDL, low density lipoprotein; SBP, systolic blood pressure.

Stepwise regression was performed to identify variables for multivariate regression. In multivariate analysis, clinic type, established diagnosis of hypertension, and SBP remained independently associated with the primary outcome.

## DISCUSSION

This study demonstrated that cardiology fellows were better at accurately assessing BP threshold for diagnosis of hypertension and hypertension treatment goals as specified by 2017 ACC/AHA hypertension guidelines than internal medicine residents even after adjusting for baseline characteristics differences between the 2 clinics. Besides the type of clinic in which the patients were assessed, the 2 other factors that were independently associated with the primary outcome were SBP and history of hypertension.

Hypertension, known to be associated with significant cardiovascular events and increased mortality,^13,14^ is an important modifiable risk factor.^15^ Several factors can lead to uncontrolled hypertension, including nonadherence to antihypertensives, unsupportive health care systems, socioeconomic barriers to accessing health care, and physician inertia.^16^ Physician, or therapeutic, inertia is defined as the failure of health care providers to initiate or advance therapy when therapeutic goals are not met and has been well described with hypertension.^17,18^ With respect to clinician-specific factors, the initial step in management of new-onset or existing hypertension is recognizing when the BP is above the recommended goal, which was the intent of this study.

The cardiology fellow clinic had a higher percentage of patients with an established diagnosis of hypertension, known ASCVD, and other cardiovascular comorbidities (congestive heart failure and atrial fibrillation), and perhaps as a result, patients had higher use of antihypertensives, aspirin, and statins. Interestingly, patients’ lipid profiles, ASCVD 10-year risk profile among patients without known ASCVD, and prevalence of diabetes and CKD were similar between the 2 clinics. Despite adjusting for the differences, clinic type was independently associated with the primary outcome. Cardiology fellows were 3.6 times more likely to accurately assess uncontrolled BP compared to internal medicine residents. We hypothesize that this difference may be attributable to increased years of specific graduate training and focused cardiovascular care in the cardiology clinic.

Established diagnosis of hypertension was also independently associated with the primary outcome, suggesting that trainees were more likely to accurately assess elevated BP in a patient with diagnosed hypertension than in a patient with an initial encounter whose BP is in the hypertensive range. In such cases, hypertension may not be the primary reason for the encounter and tends to be overlooked. We believe such encounters are missed opportunities for diagnosing new hypertensive patients and initiating measures early to prevent negative cardiovascular outcomes.

We found a strong association between SBP and the primary outcome in this study. For every 1 mmHg increase in SBP, the odds of accurate assessment of uncontrolled BP increased by 17%. In univariate analysis, isolated elevated DBP was associated with significantly lower odds of achieving the primary outcome, but this correlation was no longer significant after adjusting for SBP in the multivariate analysis. Overall, the study suggests that physicians in this study placed a greater importance on SBP than DBP when addressing hypertension, which could be explained in part by the fact that while the recommendation for an SBP goal of <130 mmHg was based on a meta-analysis by the Evidence Review Committee,^19^ data are mixed on the association of diastolic hypertension with cardiovascular outcomes,^20,21^ and the DBP goal of <80 mmHg was based on expert opinion.^3^

In this study, internal medicine residents focused on lifestyle changes, whereas cardiology fellows tended to make pharmacologic interventions. This difference is not unexpected, as cardiology clinic is often a referral clinic, and patients have likely had nonpharmacologic interventions before presenting to the clinic. Because of the small number of events, we did not adjust individual interventions for baseline differences between the 2 clinics. The higher proportion of patients with established atherosclerotic diseases and known hypertension seen in the cardiology fellow clinic was possibly associated with the higher number of medication-related interventions compared to the internal medicine resident clinic.

The overall compliance rates among the trainee physicians in this study are far lower—29.1% in the cardiology fellow clinic and 10.3% in the internal medicine resident clinic—than the compliance rates reported in the studies of previous guidelines.^6-8^ Possible causes could be lack of awareness about the current guidelines among trainees, disagreement with the 2017 ACC/AHA BP guidelines, conflicting hypertension guidelines,^22,23^ insufficient patient encounter time, hypertension not being the primary reason for patient encounter, or physician inertia. We theorize that by excluding the population that would have met JNC 8 criteria, our study identifies a population subset that is easily missed when seen in an ambulatory setting. This study suggests that the inadequate rate of compliance with BP goals by trainee physicians is a potential barrier to adequate control of hypertension in the population and represents an area for improvement.

Our study has several limitations. This retrospective study relied on information in the electronic medical records. Hence, if physicians recognized that BP was uncontrolled but did not indicate that in their notes, we could not accurately assess those encounters. Recorded race data for patients with Hispanic ethnicity were not reliable and hence not collected. ASCVD 10-year risk could not be calculated for some patients because of insufficient data, but the proportion of patients for whom ASCVD 10-year risk was not calculated was similar in both clinics. Because our study was retrospective, we were not able to assess the knowledge of the physicians regarding hypertension guidelines with a questionnaire beforehand, preventing us from definitively concluding that lack of awareness regarding the 2017 ACC/AHA guidelines among physicians contributed to the difference in assessment between the 2 clinics. Our study was a single-center study, so the results may not be generalizable to other centers. We are also limited in being able to comment on whether interventions such as focused lectures to trainees or programmed electronic medical record prompts for BP ≥130/80 mmHg would help improve BP assessment and eventually cardiovascular outcomes. These questions can be addressed by future prospective trials or quality improvement projects.

## CONCLUSION

In this retrospective study, cardiology fellows were better at identifying hypertension diagnosis thresholds and BP treatment goals in accordance with 2017 ACC/AHA hypertension guidelines compared to internal medicine residents. SBP and history of hypertension were also independently associated with accurate assessment of uncontrolled BP. We hope this study establishes a foundation for future studies examining educational tools to improve assessment and management of hypertension by trainee physicians.

## Notes

*This article meets the Accreditation Council for Graduate Medical Education and the American Board of Medical Specialties Maintenance of Certification competencies for Patient Care, Medical Knowledge, and Practice-Based Learning and Improvement.*
